# Designing Psychologically Grounded Artificial Intelligence for Supporting Bystander-Based Cyberaggression Intervention: Mixed Methods Exploratory Study

**DOI:** 10.2196/84391

**Published:** 2026-04-13

**Authors:** Jinkyung Katie Park, Pinxuan (Alina) Yu, Vignesh Krishnan, Huaye Li, Linda A Reddy, Vivek K Singh

**Affiliations:** 1 School of Computing, Clemson University, 105 Sikes Hall, Clemson, SC, United States, 1 6784466336; 2 Graduate School of Applied and Professional Psychology, Rutgers University, New Brunswick, NJ, United States; 3 Department of Computer Science, Rutgers University, New Brunswick, NJ, United States; 4 School of Communication and Informaiton, Rutgers University, New Brunswick, NJ, United States

**Keywords:** cyberaggression, bystander intervention, generative artificial intelligence, AI, large language model, theory-driven intervention, digital well-being, mental health

## Abstract

**Background:**

Cyberaggression poses a growing threat to mental health, contributing to increased distress, reduced self-esteem, and other adverse psychosocial outcomes. Although bystander intervention can mitigate the escalation and impact of cyberaggression, individuals often lack the confidence, strategies, or language to respond effectively in these high-stakes online interactions. Advances in generative artificial intelligence (AI) present a novel opportunity to facilitate digital behavior change by assisting bystanders with contextually appropriate, theory-informed intervention messages that promote safer online environments and support mental well-being.

**Objective:**

This mixed methods design study aimed to explore the feasibility of using generative AI to support bystander intervention in cyberaggression on social media. Specifically, we examined whether AI can generate effective responses aligned with established intervention strategies and how these responses are perceived in terms of their potential to de-escalate online harm and foster behavior change.

**Methods:**

We collected 1000 real-world cyberaggression examples from public social media datasets and generated bystander intervention responses using 3 distinct prompt strategies: a generic policy reminder, a baseline GPT prompt, and a theory-driven GPT prompt (AllyGPT). To evaluate the responses, we conducted computational linguistic analyses to assess their psycholinguistic features and carried out a mixed methods evaluation. Three trained coders rated each message on favorability, conversational impact, and potential to change behavior and later participated in semistructured interviews to reflect on their evaluation process and perceptions of intervention effectiveness.

**Results:**

Linguistic analyses revealed that baseline GPT responses exhibited more emotionally positive and authentic language compared to AllyGPT responses, which showed a more analytical and assertive tone. Policy reminder messages were linguistically rigid and lacked emotional nuance. Human evaluation results showed that AllyGPT responses received the highest effectiveness ratings for low-incivil cyberaggression cases in 2 dimensions (favorability and changing behavior), and baseline GPT works better for mid and high levels for all effectiveness dimensions. For medium- and high-incivility aggressions, baseline GPT responses received the highest ratings across all 3 dimensions of effectiveness (favorability, discussion-shifting potential, and likelihood of changing bullying behavior), followed by AllyGPT, with policy reminders rated lowest. Qualitative feedback further emphasized that baseline GPT responses were perceived as natural and inclusive, while AllyGPT responses, although grounded in psychological theory, were sometimes viewed as overly direct. Policy reminders were considered clear but lacked persuasive impact.

**Conclusions:**

Our work showed that designing effective AI-generated bystander interventions requires a deep sensitivity to platform culture, social context, and user expectations. By combining psychological theory with adaptive, conversational design and ongoing feedback loops, future systems can better support bystanders, delivering interventions that are not only contextually appropriate but also socially resonant and behaviorally impactful. As such, this work serves as a foundation for scalable, human-centered AI systems that promote safer online spaces and users’ mental well-being.

## Introduction

### Background

In today’s digitized world, cyberaggression has become a critical mental health concern, affecting individuals across age groups and platforms [1]. As an umbrella term encompassing behaviors such as cyberbullying, hate speech, and online harassment [2], cyberaggression carries substantial emotional and psychological weight [3]. Research consistently documents its adverse mental health impacts, including anxiety, depression, social withdrawal, and emotional dysregulation [4,5]. Meta-analyses further show that cybervictimization predicts depression, suicidal ideation, and suicide attempts among youth [6,7]. These harms extend beyond adolescence, with adults also facing targeted abuse that undermines dignity, safety, and civic participation [8-10]. Cyberaggression often co-occurs with offline aggression, amplifying psychological effects [11], and perpetrators themselves face risks such as low self-esteem, heightened aggression, and internalizing symptoms [12]. Moreover, studies indicate that vulnerable populations, such as those with existing emotional or social difficulties, may experience exacerbated distress when targeted online [13].

Recognizing these harms, scholars have examined strategies to prevent escalation and reduce impact [14-16]. One promising approach involves bystanders, witnesses to online harassment who can interrupt harmful interactions, defend victims, or report abuse [17]. Prior research has shown that online bystander intervention is often enacted through a range of behaviors beyond direct confrontation, including social norm signaling and emotional support for targets [18-21]. Research has highlighted that bystanders’ active or passive involvement can substantially influence the course and outcomes of cyberaggression by enabling real-time de-escalation and reinforcing social norms without requiring formal moderation or punitive action [20,22,23]. Yet, many remain hesitant due to uncertainty, fear of retaliation, or lack of effective language [24,25]. This work focuses on reducing barriers to action, specifically, uncertainty about how to intervene and a lack of effective language, rather than directly addressing bystanders’ underlying motivation or risk tolerance. Existing interventions often rely on static templates that fail to adapt to the nuances of diverse incidents [26]. Generative artificial intelligence (AI), particularly large language models (LLMs), offers new potential by producing scalable, human-like language for intervention [27]. However, few approaches are grounded in psychological theories of helping behavior, limiting their capacity to support intentional and effective action.

To address these gaps, this study introduces “AllyGPT,” a theory-driven LLM designed to support online bystander interventions by scaffolding context-sensitive, psychologically grounded response strategies. By integrating psychologically grounded prompt design with the generative capacities of LLMs, we aim to produce context-sensitive, linguistically appropriate responses that empower bystanders to intervene effectively. This approach advances scalable, theory-informed support for safer digital spaces and improved mental well-being.

### Related Work

#### The Role of Bystanders in Cyberaggression Intervention

Bystanders, those who witness but are not directly involved in cyberaggression, play a pivotal role in disrupting harmful dynamics [28]. Their responses vary in tone, target, and intention, ranging from aggressive actions (eg, threats and insults) to prosocial support [29,30]. Within cyberbullying, where victims often struggle to defend themselves, bystanders are particularly well studied [31]. Broadly, bystander interventions are categorized as aggressive, constructive, victim-focused, or constructive bullying-focused; aggressive strategies can escalate conflict [28,32], whereas constructive approaches, such as encouraging apologies, asking perpetrators to stop, or supporting victims with empathy or advice, are more effective in de-escalation [33-35]. Bystander interventions are uniquely embedded within ongoing interactions. Unlike post hoc moderation or long-term educational approaches, bystanders can intervene as harm emerges, offering immediate support to targets while signaling that aggressive behavior violates community norms [20,36]. This ability to reduce harm in real time and shape collective expectations makes bystander intervention a critical and complementary strategy, particularly in early-stage incidents where formal enforcement is yet to be enacted.

Large-scale discourse analyses reveal that bystanders use diverse strategies, including callouts, mocking, education, and moderation. For example, Ray et al [36] examined over 2 million Twitter and Reddit posts, finding that constructive approaches, such as educating perpetrators and mobilizing support, reduced hate speech and encouraged engagement, while mocking had little effect. Yet, intervention rates remained low, with bystanders active in only one of 6 Twitter threads and under 40% of Reddit discussions. These barriers highlight the need for scalable supports to help bystanders act effectively. Building on this, this study focuses on 5 constructive intervention subtypes, assessing both the linguistic quality (eg, tone and affect) and effectiveness (favorability, discussion shift, and behavior change) of LLM-generated responses.

#### The Potential of LLMs in Cyberaggression Bystander Intervention

LLMs have emerged as powerful tools for mitigating online harms, including cyberaggression [37,38]. Their capacity to understand and generate human-like language enables novel approaches to detecting harmful content, supporting victims, and fostering prosocial interactions [27]. For instance, Islam et al [37] proposed an LLM-based cyberbullying detection pipeline combining preprocessing, query generation, and classification for early intervention. Similarly, Vanpech et al [38] extended this approach using GPT-4 Vision to analyze images posted on Twitter and classify bullying content. These studies underscore LLMs’ potential in detection-focused interventions. Beyond detection, LLMs have been applied to bystander support and education. For example, Hedderich et al [18] developed an LLM-based chatbot platform for K-12 classrooms, enabling educators to simulate social media interactions and teach bystander strategies. Similarly, Wu et al [39] generated personalized persuasive messages aligned with strategies such as comforting, evoking, and scaffolding, showing that LLMs can adapt interventions to users’ contexts. Collectively, this work demonstrates growing interest in leveraging LLMs for intervention generation.

However, prior work has largely emphasized detection or educational use cases, leaving open questions about how LLMs can serve as in situ scaffolds for real-time bystander intervention during unfolding incidents of cyberaggression. In addition, prior studies emphasize technical alignment over psychological grounding [40]. Without integration of established bystander theories, LLM-generated interventions risk overlooking the cognitive and emotional processes driving real-world decisions. To address this gap, our study examines 5 intervention strategies rooted in bystander theory, using prompt engineering to generate responses that are linguistically appropriate, context aware, and psychologically informed. Therefore, we ask, “How can generative AI be designed to provide psychologically grounded, context-sensitive suggestions for bystanders intervening in cyberbullying scenarios?”

### Study Purpose

The primary goal of this study was to explore the potential of generative AI to support scalable, psychologically grounded bystander intervention responses to cyberbullying. To meet this goal, this study aimed to address 3 objectives:

- To design prompt strategies informed by psychological theories of bystander behavior (eg, the 5 bystander intervention responses) for generating constructive, context-sensitive intervention messages using LLMs
- To evaluate the quality and effectiveness of AI-generated bystander intervention responses (eg, standard policy reminder, baseline GPT, and theory-driven GPT) across dimensions such as linguistic characteristics, favorability, potential for altering discussion and likelihood of changing aggressive behavior
- To generate design guidelines and theoretical insights for building safe, scalable, and psychologically informed AI systems that empower bystanders to intervene in harmful online interactions

We addressed the following research questions (RQs):

- RQ1: How do bystander intervention responses generated by (1) standard policy reminder, (2) baseline GPT, and (3) theory-driven AllyGPT differ in terms of psycholinguistic characteristics (eg, authoritative, emotional tone)?
- RQ2: How effective are bystander intervention responses generated by (1) standard policy reminder, (2) baseline GPT, and (3) theory-driven AllyGPT?

## Methods

### Study Overview

We collected publicly available datasets of real-world cyberaggression and developed 3 bystander intervention strategies reflecting varying levels of specificity and psychological framing. Using these strategies, we generated intervention responses. To address RQ1, we analyzed their linguistic and psychological characteristics with the Linguistic Inquiry and Word Count (LIWC) tool, assessing features such as tone, cognitive processes, emotionality, and authenticity. To address RQ2, we conducted a human-centered evaluation in which three trained external coders rated each response on (1) *perceived favorability*, (2) *potential to shift the discussion*, and (3) *likelihood of changing aggressive behavior*. Coders then participated in semistructured interviews to elaborate on their evaluation criteria, preferences, and reflections. Quantitative and qualitative analyses were conducted to compare psycholinguistic features and perceived effectiveness across strategies.

### Ethical Considerations

The study was approved by Rutgers University’s Institutional Review Board (Study ID: Pro2024001658). All coders provided informed consent prior to participation and were informed of their rights, including the ability to withdraw at any time without penalty. Given the potentially sensitive nature of the materials, coders were informed in advance about the content and provided with contact information for support resources if participation caused discomfort. Interview recordings and transcripts were deidentified prior to analysis, and all study data were stored on an institutional review board–approved, secure cloud server with access restricted to authorized research personnel only.

### Data Collection

#### Cyberbullying Dataset

We collected 1000 cyberaggression examples from the 2 sources [41,42]. Of 1000 cyberaggression examples, 200 were collected from the dataset created by Huang et al [40], while 800 were collected from the dataset created by ElSherief et al [41].

#### Bystander Intervention Strategies

##### Overview

We had three bystander intervention strategies: (1) standard policy reminder, (2) baseline GPT, and (3) AllyGPT. Each condition supports different mechanisms that enable bystanders to progress toward intervention. Policy reminders act as normative interventions, signaling collective standards that legitimize action without direct confrontation [21]. Baseline GPT responses provide generic intervention scaffolds that reduce barriers to knowing how to act [24,43,44]. AllyGPT responses are theory-aligned, context-sensitive interventions that scaffold empathy, perspective-taking, or de-escalation to address cognitive and affective barriers to action [44-47]. Below, we explain how we designed our bystander intervention strategies.

##### Strategy 1: Standard Policy Reminder

The following standard policy reminder was taken from the Reddit platform [48] , and the same reminder was applied as an intervention message to all cyberbullying examples:

*We do not tolerate the harassment, threatening, or bullying of people on our site; nor do we tolerate communities dedicated to this behavior.*

##### Strategy 2: Baseline GPT

For the baseline GPT strategy, we used the following prompt to generate bystander intervention responses using the GPT-4o model. The same prompt was used to generate intervention responses for all cyberbullying examples: “Generate a bystander intervention response to the comment.”

##### Strategy 3: AllyGPT

To tailor intervention messages to the specific context of each cyberaggression scenario, we designed a structured prompting framework in which GPT first assessed the level of incivility and direction of aggression. With the 2 dimensions, a decision tree (see below for details) was used to select the appropriate combination of bystander intervention strategies. The same prompt was used with GPT-4o to generate intervention responses for all cyberaggression examples. The full mapping between the aggression context and intervention strategy is detailed below.

### The Theoretical Ground and Design of AllyGPT

#### The 5 Bystander Strategies Designed Based on Theories and/or Empirical Evidence in Psychology

We implemented 5 theoretically and empirically grounded strategies for prompting LLM-generated bystander intervention responses. Each strategy was derived from psychological literature and operationalized through specific prompts in the form of “dos and don’ts” to guide GPT responses. Table 1 presents a brief overview of the definition, psychological basis, and prompting guidelines for each bystander intervention (see Multimedia Appendix 1 for full details).

**Table 1.** Definition, psychological ground, and prompt of the 5 bystander intervention strategies.

Strategy	Definition	Psychological basis	Prompting guidelines
Calling out the aggressive behavior	Explicitly points out the harm or injustice in the aggressive behavior and may question or ask the perpetrator to stop.	Defended victims report higher self-esteem and less distress than those without defenders [49].	*Do*: Focus on the content of the aggression. *Don’t*: Avoid swearing or personal insults.
Correcting misinformation	Offers factual corrections using evidence, statistics, or historical context to challenge harmful or misleading claims.	Educative interventions are shown to de-escalate online discourse and create long-term learning moments [36,50].	*Do*: Focus on facts and the behavior, not the person. *Don’t*: Avoid engaging in back-and-forth debate.
Validating and expressing empathy	Acknowledges the victim’s emotional experience and offers emotional support.	Empathic responses increase self-esteem and coping and mitigate the negative effects of aggression [51-53].	*Do*: Validate the victim’s specific statements and show care and understanding. *Don’*t: Avoid making assumptions or escalating the conflict.
Advising disengagement	Encourages the victim to leave the conversation or disengage from the harmful interaction to prioritize their well-being.	Supportive disengagement can improve emotional adjustment and empower the victim without implying blame [54,55].	*Do*: Empower the victim to prioritize self-care and autonomy. *Don’t*: Avoid commands or framing exit as weakness.
5.Redirecting the discussion	Shifts the conversation away from the aggression topic to defuse tension and divert attention from the perpetrator.	Topic redirection minimizes exposure to harm and helps de-escalate aggression [56].	*Do*: Introduce a new, unrelated topic to shift attention. *Don’t*: Avoid acknowledging or directly responding to the aggressive content.

#### Decision Tree for Selecting Strategies and Drafting Bystander Intervention Message

To generate bystander intervention responses, we prompted AllyGPT to first assess each aggressive message based on 2 dimensions: incivility level (low, moderate, or high) and direction (group targeted vs individual targeted). These dimensions are grounded in bystander intervention theory and empirical research on cyberaggression, which identifies severity appraisal and target identifiability as primary determinants of whether and how bystanders intervene. Bystander intervention theory conceptualizes intervention as a sequential process in which individuals must first interpret an event as sufficiently serious to warrant action and to assume responsibility [44], while empirical studies show that incident severity strongly predicts intervention likelihood and interacts with empathy and responsibility attribution [45]. Direction further shapes responses by influencing emotional salience, perceived responsibility, and social visibility: person-targeted aggression elicits clearer moral judgments and stronger empathic engagement, whereas group-targeted harm more often leads to diffusion of responsibility and reduced effectiveness of individualized responses [46]. Prior work also suggests that empathy-focused strategies are more effective in lower-severity contexts, while assertive or confrontational approaches are more appropriate in higher-severity or public settings [47]. Together, these insights informed AllyGPT’s selection and conditional combination of intervention strategies to generate context-sensitive bystander responses. Below, we present the prompt encoding this decision logic.

**Figure 1.**
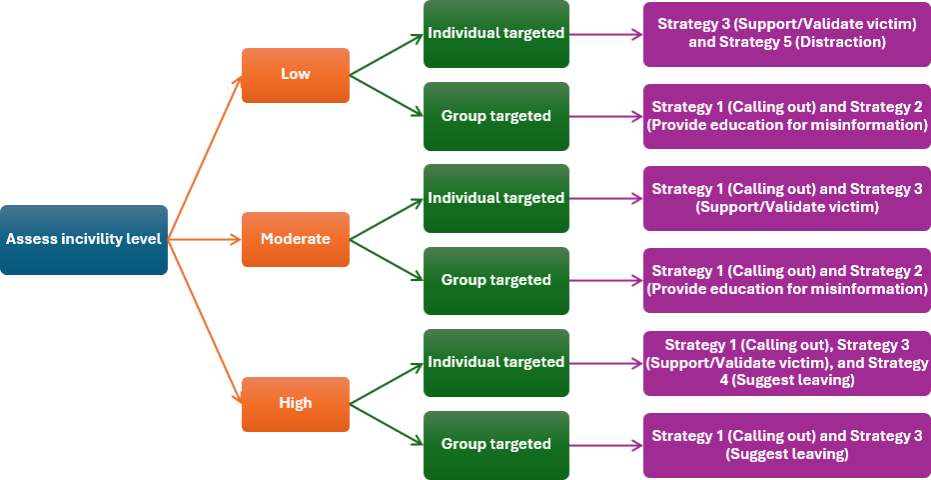
Visualized decision tree to generate bystander intervention responses.

1. Assess the *incivility level* of the aggressive message:
  - If the message does not contain profane language such as name-calling, swearing, or threatening content, then the incivility level of the message will be *low*.
  - If the message contains name-calling, slang, or swearing content but does not include threats of violence or aggression, then the incivility level of the message will be *moderate*.
  - If the message contains threats, violence, or aggression, then the incivility level of the message will be *high*.
2. Then assess the *direction* of the bullying:
  - If the message is more general and not person-specific (eg, vague insults, general hateful comments, and directed at a group but not an individual), then the direction is *group targeted*.
  - If the message is person-specific and targets an individual (eg, quoting a certain user’s ID or name and personal attacks), then the direction is *individual targeted*.
3. Consider both the incivility level and direction to select and combine the most effective strategies, following the rationale that:
  - If the message contains low to moderate incivility, consider educational approaches and distract attention; otherwise, prioritize removing the potential victim from further harm by suggesting leaving; and
  - If aggression is person specific and targets an individual, prioritize emotional support for the victim and removing the victim from further harm by suggesting leaving; otherwise, focus on education for misinformation and distracting attention.
4. Refer to the following suggestion for bystander intervention is made for different types of cyberaggression statements (Figure 1).

Through the above process, we generated 3 responses for each of the 1000 cyberaggression scenarios, resulting in a total of 3000 bystander responses (1000 per strategy) across the three intervention approaches: (1) standard policy reminder, (2) baseline GPT, and (3) AllyGPT.

### Evaluation Metrics

#### Linguistic Differences

To evaluate the linguistic differences across bystander intervention responses generated by 3 different strategies, we used the LIWC [57] to extract several linguistic features from the bystander responses. The features we extracted in this study included word count (total number of words), words per sentence (average sentence length), analytical thinking (degree of logical and formal thinking), authenticity (extent of honest and genuine expression), positive tone (prevalence of positive emotional language), negative tone (prevalence of negative emotional language), and netspeak (use of internet-specific shorthand and casual online expressions, eg, “lol” and “brb”).

#### Perceived Effectiveness Measures

The effectiveness of the bystander intervention messages was evaluated using three variables: (1) *perceived favorability*, (2) *discussion shift*, and (3) *behavior change*. Three trained coders independently rated each bystander intervention response on 3 effectiveness dimensions using a 5-point Likert-type scale (1=strongly disagree and 5=strongly agree). The first dimension, *perceived favorability,* assessed the extent to which a response would likely be viewed positively by other social media users (mean 3.88, SD 0.88). The second dimension, *discussion shift*, evaluated the potential for the response to redirect the conversation toward a more positive, objective, and/or less antagonistic tone (mean 3.95, SD 0.81). The third dimension, *behavior change*, measured the perceived likelihood of the response influencing the bully’s or agitator’s future online behavior (mean 3.45, SD 0.86).

Each coder was responsible for evaluating bystander intervention responses associated with all 1000 cyberaggression scenarios, ensuring exposure to all 3 intervention strategies. To achieve this, the intervention responses were divided into 3 batches so that each coder rated one-third of the responses from each strategy. For example, coder 1 evaluated strategy 1 responses from batch 1 (aggressive messages 1‐333), strategy 2 responses from batch 2 (messages 334‐666), and strategy 3 responses from batch 3 (messages 667‐1000; Table 2). This approach ensured that each coder rated the effectiveness of intervention responses across all 3 strategies while covering the full set of 1000 cyberaggression examples. Table 3 presents the example aggressive messages, bystander responses generated from the 3 strategies, and coder ratings of the 3 effectiveness variables.

**Table 2.** Distribution of coding assignments across 1000 cyberaggression examples, ensuring each coder evaluated responses from all 3 bystander intervention strategies.

Batches	Bystander response strategy 1 (n=1000)	Bystander response strategy 2 (n=1000)	Bystander response strategy 3 (n=3000)
Batch 1: aggressive messages 1‐333	Coder 1	Coder 3	Coder 2
Batch 2: aggressive messages 334‐666	Coder 2	Coder 1	Coder 3
Batch 3: aggressive messages 667‐1000	Coder 3	Coder 2	Coder 1

**Table 3.** Example aggressive messages, bystander responses generated by the 3 intervention strategies, and the effectiveness scores rated by the coders (in the order of favorability or discussion shift and behavior change).

Aggressive message and intervention strategies	Bystander responses	Effectiveness scores
Why are black fathers and Shaq’s free throw attempts the same? They’re always missing		
Policy reminder	“We do not tolerate harassment, threatening, or bullying of people on our site; nor do we tolerate communities dedicated to this behavior.”	4/4/3
Baseline GPT	“It’s important to spread positivity and respect in our community. Racist or derogatory jokes are not only offensive but also foster a harmful environment. Let’s aim for constructive and respectful interaction instead.”	4/4/3
AllyGPT	“Such stereotyping is decidedly offensive and it’s not okay to continue this conversation in a disrespectful manner. To the person affected by this, please know that your value is not determined by such uninformed comments. This conversation is becoming progressively more toxic, and it might be beneficial for you to step away for a while.”	5/5/5
mim torse lottttttta ugly white people here . are you mad at the immigrants cuz they sexy		
Policy reminder	“We do not tolerate harassment, threatening, or bullying of people on our site; nor do we tolerate communities dedicated to this behavior.”	4/4/3
Baseline GPT	“There’s definitely room for diverse opinions here, but let’s aim to maintain respect for all members and avoid generalizations. Beauty, like attractiveness, is subjective and varies greatly between individuals, cultures, and societies. Let’s remember, every ethnicity has its own unique charm and attractiveness. Regarding the immigration issue, it’s a complex topic and it’s possible to have a meaningful discussion without degrading each other.”	5/5/4
AllyGPT	“The comment you’re making is disrespectful and propagates unfounded stereotypes. Instead of focusing on differences, let’s embrace our shared humanity. Each person is unique, and beauty is subjective and multi-faceted. In regards to immigration, it’s important to remember that people immigrate for diverse reasons, and making assumptions based on their appearance is unhelpful and can spread misinformation.”	4/4/4

Three graduate students in psychology (2 doctoral and one master’s) were trained as independent raters. Training involved a structured, multistep process combining group instruction, guided practice, and independent rating. Coders were first introduced to the study purpose, research questions, and their own experiences with social media and cyberaggression. They were then provided with a detailed coding manual containing sample scenarios, example ratings, and rationales for the 3 effectiveness dimensions (favorability, discussion shift, and behavior change).

As a group, coders practiced rating 3 scenarios, followed by discussion and feedback to build consensus. They next completed independent practice ratings on 3 additional scenarios, which were reviewed and discussed to refine consistency. Coders were also trained on the Qualtrics survey platform, including rating procedures and response scales. To establish intercoder reliability, each independently rated 25 additional scenarios; consensus agreement of 80% or greater was required before proceeding to the full study (1000 scenarios per coder and 3000 total).

A 1-hour booster session was conducted midway through coding to address procedural questions, clarify operational definitions, and review rating criteria. Coders jointly discussed 2 example responses, examining features linked to higher versus lower ratings across the dimensions. Intercoder agreement was computed using a group consensus method. Individual coder ratings for each of the 25 scenarios and the 3 effectiveness dimensions (perceived favorability, discussion shift, and behavior change) were compared to the group mean ratings. Agreement was considered achieved when coder ratings fell within one point of the group mean. For the 3 effectiveness dimensions, the Krippendorff α was 0.73 for favorability, 0.84 for discussion shift, and 0.89 for behavior change.

#### Semistructured Interview

After completing the survey, each coder participated in a separate semistructured exit interview conducted by 2 researchers. Through the interviews, we explored coders’ views on the effectiveness of responses generated by the 3 strategies, their overall coding experience, and any questions about the study. To aid recall, coders were reminded of the rating scales and were shown their average scores across the 3 effectiveness dimensions. They also reviewed 2 example aggression scenarios with responses from all strategies: one where baseline GPT outperformed AllyGPT and another where AllyGPT outperformed baseline GPT across all dimensions. Examples were selected to ensure consistent trends across dimensions, randomly sampled from different coding batches, and paired with the corresponding policy reminder. Interviews lasted about 50 minutes, were audio recorded, transcribed, and deidentified. Coders received compensation for their participation.

### Data Analysis Approach

For RQ1, we used a set of *t* tests to compare the linguistic characteristics of bystander intervention responses generated by baseline GPT and AllyGPT. An α of .05 was used to determine the statistical significance of the mean difference between the 2 intervention strategies. As all policy reminders consisted of identical messages across all cases, their linguistic features (eg, word count and positive words) exhibited zero variance, and they were excluded from statistical analyses comparing mean differences across strategies. Descriptive statistics for standard policy reminders are reported separately to provide a benchmark, while statistical comparisons were conducted between AllyGPT and baseline GPT, where variation in generated responses allowed for meaningful analysis.

To address RQ2, we used a set of ANOVA and *t* tests to compare the average perceived effectiveness rating across (1) 3 different bystander intervention strategies, (2) incivility level, and (3) the direction of aggression (individual vs group). An α of .05 was used to determine the statistical significance of the mean difference across the 3 intervention strategies. Next, we conducted a series of robust general linear models using heteroskedasticity-consistent SEs to examine the main and interaction effects of intervention strategy (policy reminder, baseline GPT, and AllyGPT), incivility level (low, moderate, and high), and direction of aggression (group targeted vs individual targeted) on the 3 outcome measures. Pairwise contrasts were conducted to compare strategy conditions, and significant interactions were further probed using simple effects analyses.

Finally, the interview data were qualitatively analyzed using a thematic analysis approach. A single researcher conducted the primary qualitative analysis using an inductive thematic approach. Transcripts were read iteratively to build familiarity, after which initial codes were generated to capture patterns in participants’ reflections on response effectiveness, cross-strategy comparisons, and coding challenges. Codes were refined through constant comparison and analytic note-taking, then synthesized into higher-level themes. Identified codes and themes were reviewed with the other authors as an analytic quality check to assess coherence and clarify interpretations. As coding was not independently replicated, no formal intercoder disagreement resolution was used. We refer to individual coders using identifiers (eg, coder 1) when presenting quotes in the qualitative findings.

## Results

### Descriptive Statistics

The final dataset consisted of 996 cyberaggression scenarios and their corresponding bystander responses, all of which were evaluated by the 3 human coders. Four (0.04%) scenarios were excluded through listwise deletion due to missing values; given the small number of cases removed (<1% of the dataset), this exclusion is unlikely to have biased the results. Among the 996 cyberaggression scenarios, 4.6% (n=46) of the cases were classified as low incivility, 48.3% (n=481) were classified as moderate, and 47.1% (n=469) were classified as high. Regarding the target of bullying, 75.7% (n=754) of the cases were group targeted, while 24.3% (n=242) were individual targeted (Table 4). These distributions indicate that the majority of cyberaggression cases in the dataset involved moderate to high levels of incivility and were more often directed at groups rather than individuals. Example responses generated by the 3 bystander intervention strategies by the incivility level and the direction of aggression are shown in Multimedia Appendix 2.

**Table 4.** The distribution of the cyberbullying example dataset in terms of the level of incivility and the direction of incivility.

Level of incivility	Group targeted (n=754, 76%), n (%)	Individual targeted (n=242, 24%), n (%)	Total (N=996, 100%), n (%)
Low incivility	34 (3.4)	12 (1.2)	46 (4.6)
Medium incivility	341 (34.2)	140 (14.1)	481 (48.3)
High incivility	379 (38.1)	90 (9)	469 (47.1)

### RQ1: Linguistic Differences Among the 3 Bystander Intervention Strategies

To assess linguistic differences across intervention strategies, we conducted independent samples *t* tests comparing messages generated by the baseline GPT and AllyGPT using LIWC 2022 variables. We used fixed-format policy reminders as a linguistic benchmark, which were excluded from *t* tests due to their lack of variability (Table 5).

**Table 5.** Linguistic characteristics of bystander intervention messages generated by the 3 different strategies (policy reminder, baseline GPT, and AllyGPT).

Characteristics	Policy reminders, mean	Baseline GPT, mean (SD)	AllyGPT, mean (SD)	*t* test (df)	*P* value	Direction
WC^a^	25 (0)	65.56 (21.71)	46.61 (11.69)	24.29 (1532.06)	<.001	Policy<AllyGPT<baseline GPT
WPS^b^	25 (0)	16.65 (4.12)	13.63 (2.91)	18.89 (1792.75)	<.001	Policy<AllyGPT<baseline GPT
Analytic	28.63 (0)	37.43 (21.66)	44.41 (26.20)	−6.49 (1927.67)	<.001	Policy<baseline GPT<Ally GPT
Authentic	85.17 (0)	45.88 (27.81)	33.40 (27.55)	10.07 (1996)	<.001	AllyGPT<baseline GPT<policy
Negative	12 (0)	2.38 (2.34)	4.66 (3.53)	12.68 (1996)	<.001	Baseline GPT<AllyGPT<policy
Positive	0 (0)	6.55 (3.43)	4.65 (3.25)	−17.01 (1732.28)	<.001	Policy<AllyGPT<baseline GPT
Netspeak	0 (0)	0.09 (0.51)	0.04 (0.44)	2.43 (1995.29)	.02	Policy<AllyGPT<baseline GPT

a WC: word count.

b WPS: word per sentence.

All 996 messages produced using policy reminders were identical, yielding a fixed *word count* of 25 and *words per sentence* of 25, with no variability across messages. In contrast, messages generated by the 2 GPT-based strategies demonstrated significantly greater linguistic variation. An independent samples *t* test (Welch) comparing baseline GPT and AllyGPT revealed that bystander intervention responses generated by baseline GPT were significantly longer than those generated by AllyGPT. Similarly, baseline GPT produced messages with a greater mean words per sentence compared to those generated by AllyGPT. Overall, the policy reminder messages were uniform and minimal in length and complexity, while both GPT-based strategies generated more linguistically varied responses. Among the generative approaches, the baseline GPT produced messages that were longer and more syntactically complex than those generated by the AllyGPT strategy.

For the *analytic thinking* score, baseline GPT messages had significantly lower means compared to AllyGPT, with Welch correction for unequal variances. This means that AllyGPT-generated bystander intervention responses are in a more structured and logical language than the baseline GPT. Both GPT-generated strategies were more analytical than the policy reminders, which scored even lower, reflecting their templated and rigid format. In contrast, *authentic language* was more prevalent in baseline GPT than in AllyGPT responses, assuming equal variances, suggesting that the baseline GPT produced more personally expressive messages. Notably, policy reminders had the highest authenticity score among all strategies, reflecting their formalized stance and declarative tone.

For *positive emotional tone*, baseline GPT responses scored higher than those from our GPT. Policy reminders, in contrast, scored zero on positive emotion, reflecting their strictly normative stance. Conversely, *negative emotional tone* was more prominent in AllyGPT messages than in baseline GPT, with unequal variances assumed. The policy reminder messages showed the highest negative emotional tone, indicating elevated levels of negative emotion due to their emphasis on prohibition and sanctions. Policy reminders scored much lower, aligning with their strict and emotionally neutral or negative framing. Finally, the use of *netspeak* (eg, online slang) was significantly higher in baseline GPT than in AllyGPT. Policy reminders contained no netspeak, consistent with their formal and standardized tone.

Together, these findings highlight distinct linguistic signatures of each intervention strategy. Baseline GPT responses tended to be emotionally positive and authentic, while AllyGPT emphasized structured reasoning and a more assertive tone. The fixed policy reminders, although consistent and easily recognizable, lacked emotional nuance and personalization, underscoring the value of generative AI for producing more context-aware and psychologically resonant interventions. Table 5 presents the mean difference and the direction of the difference in psycholinguistic characteristics across the 3 bystander interventions.

### RQ2: Perceived Effectiveness of Bystander Interventions

#### Quantitative Results

##### Main Effects of Intervention Strategies on Perceived Effectiveness

###### Perceived Favorability

A one-way Welch ANOVA was conducted to examine differences in perceived favorability across the 3 intervention strategies: standard policy reminders, baseline GPT-generated responses, and AllyGPT-generated responses. The analysis revealed a significant effect of intervention type on perceived favorability (*P*<.001). Post hoc Games-Howell comparisons indicated that all pairwise differences were statistically significant (*P*<.005). The mean favorability ratings were highest for the baseline GPT responses, followed by AllyGPT responses and standard policy reminders.

###### Potential for Altering the Discussion

A one-way Welch ANOVA revealed significant differences in how effective interventions were perceived at shifting discussions. Baseline GPT responses again received the highest ratings, followed by AllyGPT, with policy reminders receiving the lowest ratings (*P*<.001). Post hoc comparisons using the Games-Howell correction indicated all comparisons reached statistical significance (*P*<.005).

###### Likelihood of Changing Aggressive Behavior

A one-way Welch ANOVA indicated a statistically significant difference in perceived effectiveness in changing bullying behavior among the 3 intervention types: standard policy reminder, baseline GPT-generated responses, and AllyGPT-generated responses (*P*<.001). Post hoc Games-Howell tests revealed that all pairwise differences were statistically significant (*P*<.001). Table 6 summarizes the mean difference and the direction of difference.

**Table 6.** Three effectiveness variables across the 3 intervention strategies.

Variables	Policy reminder (n=996), mean (SD)	Baseline GPT (n=996), mean (SD)	AllyGPT (n=996), mean (SD)	Welch ANOVA		Post hoc test
				*F* test (df)	*P* value	
Favorability	3.50 (0.50)	4.14 (0.99)	3.98 (0.92)	219.28 (2, 1792.02)	<.001	Baseline GPT>AllyGPT>policy reminder
Altering discussion	3.81 (0.39)	4.16 (0.96)	3.89 (0.90)	58.30 (2, 1682.03)	<.001	Baseline GPT>AllyGPT>policy reminder
Changing behavior	3.06 (0.29)	3.83 (1.00)	3.45 (0.91)	328.77 (2, 1542.98)	.001	Baseline GPT>AllyGPT>policy reminder

### Main Effects of the Aggression Context on Perceived Effectiveness

#### Incivility Level and the Perceived Effectiveness of Bystander Responses

A one-way ANOVA indicated that there was no statistically significant difference in mean values for perceived favorability, effectiveness in altering the discussion, and effectiveness in changing bullying behavior across intervention messages generated for high-level, medium-level, and low-level aggressive messages (Table 7).

**Table 7.** Three outcome variables by incivility level.

Variables	Low (n=138), mean (SD)	Moderate (n=1443), mean (SD)	High (n=1407), mean (SD)	ANOVA	
				*F* test (df)	*P* value
Favorability	3.88 (0.76)	3.88 (0.87)	3.86 (0.89)	0.202 (2, 2985)	.81
Altering discussion	3.94 (0.67)	3.96 (0.81)	3.95 (0.82)	0.09 (2, 2985)	.90
Changing behavior	3.46 (0.73)	3.44 (0.88)	3.45 (0.85)	0.06 (2, 2985)	.93

#### Direction of Aggression and the Perceived Effectiveness

Independent-samples *t* tests revealed significant differences in the perceived effectiveness of intervention messages depending on whether the aggression was group targeted or individual targeted. Responses addressing group-targeted aggression were rated as significantly more favorable than those addressing individual-targeted bullying (*P*<.001). Similarly, interventions for group-targeted bullying were seen as more effective in altering discussions than for individual-targeted bullying (*P*<.001). Finally, messages addressing group-targeted bullying were rated as more effective in changing bullying behavior than those addressing individual-targeted bullying (*P*=.001; Table 8).

**Table 8.** Three outcome variables by direction of aggression.

Variables	Group (n=2262), mean (SD)	Individual (n=726), mean (SD)	*t* test (df)	*P* value
Favorability	3.92 (0.84)	3.74 (0.95)	4.46 (1115.39)	<.001
Altering discussion	3.99 (0.77)	3.85 (0.90)	3.56 (1090.78)	<.001
Changing behavior	3.48 (0.83)	3.35 (0.93)	3.20 (1115.36)	.001

### Interaction Effects of Bystander Intervention and Aggression Context on Perceived Effectiveness

### Interaction Effects of Bystander Strategy and Incivility Level on the Perceived Effectiveness

#### Overview

We conducted a series of robust general linear models with heteroskedasticity-consistent SEs to examine the main and interaction effects of strategy (standard policy reminder, baseline GPT, and AllyGPT) and incivility level (low, moderate, and high) on 3 outcome measures. Pairwise contrasts were used to compare strategy conditions, and significant interactions were further examined using simple effects analyses. Overall, strategy type mattered more than incivility level. Across all outcomes, GPT-generated responses (both baseline and AllyGPT) were consistently rated higher than policy reminders. Incivility level alone did not significantly influence ratings, and very few strategy ×incivility interactions emerged. The only notable exception was that baseline GPT responses were perceived as more effective in changing behavior when paired with moderately incivil messages.

#### Perceived Favorability

There was a significant main effect of strategy type, in which GPT-based responses were rated as more favorable than policy reminders. Incivility level and interaction effects were not significant (Table 9).

**Table 9.** General linear model results for the strategy and incivility level interaction.

Variables	Main effect of the strategy	Main effect of incivility	Interaction effect (*B*)	Key contrasts (*B*)
Favorability	GPTs>policy (*P*<.005)	—^a^	—	AllyGPT (+0.48), baseline GPT (+0.41) versus policy
Altering discussion	—	—	—	GPT responses are directionally higher
Changing behavior	GPTs>policy (*P*<.005)	—	Baseline GPT×moderate incivility (+0.30, *P*=.03)	AllyGPT (+0.61), baseline GPT (+0.52) versus Policy

a Nonsignificant.

#### Altering Discussion

No significant main or interaction effects were observed. Although GPT-based responses were directionally more positive, the differences were not statistically significant.

#### Behavior Change

Both baseline GPT and AllyGPT responses were rated as more effective than policy reminders. A small interaction was observed, with baseline GPT showing an added advantage under moderate incivility.

**Figure 2.**
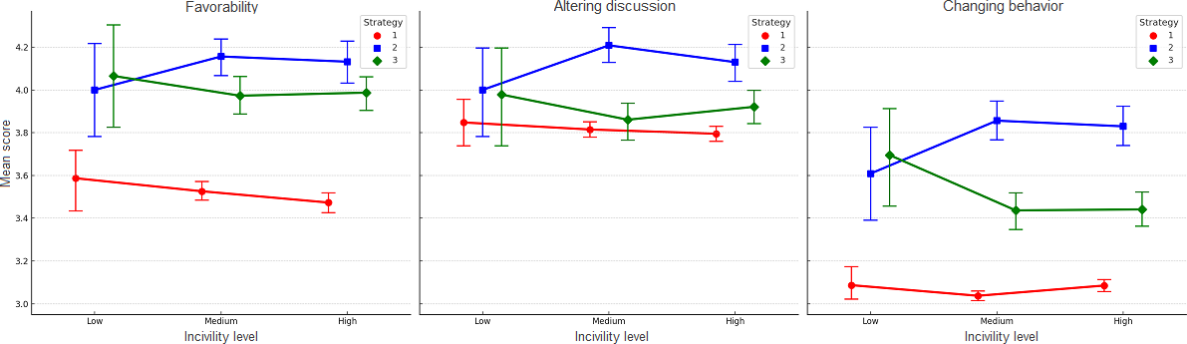
Interaction effects of strategy and incivility level on bystander intervention ratings across 3 outcome variables: favorability, altering discussion, and changing behavior. Mean scores and 95% CIs are plotted for each strategy (red=policy reminder, blue=baseline GPT, and green=AllyGPT) at each incivility level (low, medium, and high).

Figure 2 visualizes the interaction effects of strategy and incivility level on bystander intervention ratings across 3 outcome variables. Although the interaction effect was not statistically significant, exploratory pairwise comparisons indicated a pattern in which AllyGPT responses were rated more favorably and more effective for behavior change than baseline GPT responses at low levels of incivility. These differences were not observed at moderate or high levels of incivility.

### Interaction Effects of Intervention Strategy and Direction of Aggression

#### Perceived Favorability

GPT-based responses (both baseline and AllyGPT) were rated more favorably than policy reminders. Responses addressing person-targeted aggression were rated less favorably than those addressing group-targeted bullying. No significant interactions were observed (Table 10).

**Table 10.** General linear model results for the strategy and direction of aggression interaction.

Variables	Main effect of the strategy	Main effect of direction	Interaction effect (*B*)	Key contrasts (*B*)
Favorability	GPTs>policy (*P*<.001)	Person<group (*P*<.001)	—^a^	Baseline GPT (+0.63), AllyGPT (+0.51) versus policy
Altering discussion	GPTs>policy (*P*<.001)	—	—	Baseline GPT (+0.39), AllyGPT (+0.12) versus policy
Behavior change	GPTs>policy (*P*<.001)	—	AllyGPT×person targeted (−0.26, *P*=.001)	Baseline GPT (+0.79), AllyGPT (+0.45) versus policy

a Nonsignificant.

#### Altering Discussion

Baseline GPT responses were rated as most effective in shifting discussions, followed by AllyGPT, with policy reminders rated lowest. The direction of bullying did not significantly influence ratings. No interaction effect was found.

#### Changing Aggressive Behavior

Both baseline GPT and AllyGPT responses were perceived as more effective than policy reminders. A significant interaction emerged: AllyGPT responses were rated as less effective in changing behavior when applied to person-targeted bullying compared to group-targeted bullying. Other interactions were statistically not significant.

**Figure 3.**
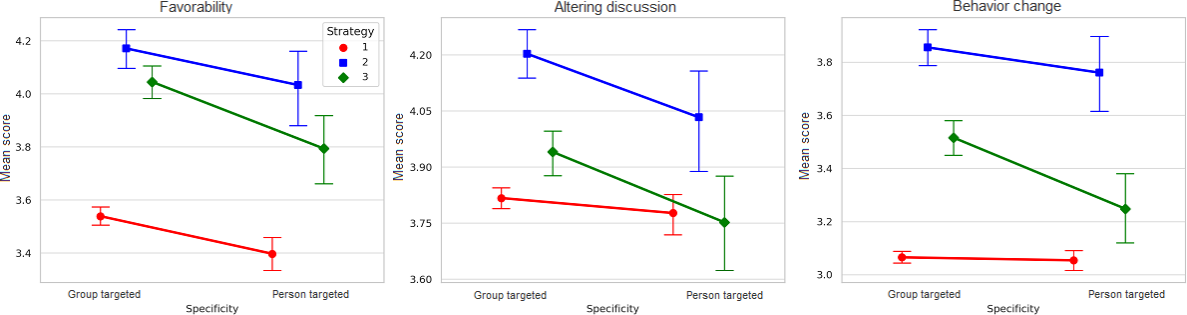
Interaction effects of strategy type (red=policy reminder, blue=baseline GPT, and green=AllyGPT) and direction (group targeted vs person targeted) on 3 perceived outcomes of bystander intervention messages: favorability, altering discussion, and behavior change, with error bars indicating 95% CIs.

Figure 3 visualizes the interaction effects of strategy type and specificity on 3 outcome variables.

### Qualitative Insights Into the Perceived Effectiveness of Intervention Strategies

#### Perceived Favorability of Intervention Strategies

The semistructured interviews with the coders provided nuanced perspectives on the perceived effectiveness of 3 different intervention strategies. First, the coders consistently described the standard policy reminder as robotic and impersonal. While these messages were generally viewed as inoffensive and safe, their lack of contextual adaptation made them feel generic. However, coders noted that this very standardization helped prevent the messages from seeming intrusive or out of place within online conversations: “It’s robotic but doesn’t feel like it’s coming out of nowhere. It kind of works because it doesn’t assume too much” (coder 1).

Baseline GPT responses were received more favorably than policy reminders due to their balance of specificity and neutrality. Coders noted that these responses avoided being too generic while also refraining from making direct or accusatory assumptions about the bully’s intent: “*They don’t first attack the bully. They kind of try to educate the bully. It’s not a very aggressive message*” (coder 2). The tone of these responses was described as gentle, constructive, and inclusive. One coder appreciated the frequent use of collective language (eg, “we” and “let’s”), which conveyed a shared commitment to respectful communication within online communities: “It’s using a lot of ‘we’ language... and I find that more favorable for viewing” (coder 1).

Coders described the responses generated by the AllyGPT prompting as the most specific, often incorporating detailed psychological framing and references to the nature of the aggression. While this specificity occasionally added persuasive depth and a human-like tone, it also introduced potential drawbacks. In cases where the response identified specific problematic content or implied motive (eg, racial bias), coders noted that such directness might provoke further defensiveness or hostility from bullies: “It’s kind of diving deeper and assuming the comment writer’s thought processes... It feels like there’s a little more of an argumentative tone*”* (coder 3). Although coders acknowledged that firmer messages could resonate in highly aggressive scenarios, they also emphasized that overt specificity or presumed interpretation could reduce the intervention’s acceptability, especially when assumptions do not align with the aggressor’s perceived intent.

#### Effectiveness of Altering the Discussion in More Positive Ways

Human coders widely agreed that the GPT-generated responses, particularly those from both baseline GPT and AllyGPT prompts, were generally more effective in shifting conversations toward a more positive and objective tone compared to the standardized policy reminders. While coders acknowledged that the standard policy reminder emphasized the importance of respectful communication, they felt this response “points out the need for more objectivity, but doesn’t really lead to a positive direction” (coder 1). As a result, they did little to steer the conversation in a constructive direction.

In contrast, coders perceived the baseline GPT responses as more successful in nudging conversations toward civility. These responses not only emphasized objectivity and respectful tone but also offered an alternative path for engagement, such as refocusing the discussion on shared understanding or mutual respect. This dual approach, highlighting a problem while proposing a constructive next step, was particularly appreciated by the coders. They also emphasized that the emotionally neutral and nonconfrontational tone used in these responses helped defuse tension. This approach, in their view, increased the likelihood that others would respond positively.

*It emphasizes that we’re all different... it redirects the conversation in a more positive and objective way. [...] Generally, people are more receptive when things are not aggressive or sounding too emotional*

[Coder 2]

Coders noted that the customized responses generated by the AllyGPT model often provided in-depth reasoning for why a comment was harmful while stressing the importance of respectful dialogue and the responsibility of maintaining safe social spaces. However, the more assertive tone and direct confrontation of aggressive behavior raised concerns among coders. Some felt that these responses, while well-intentioned, could come across as accusatory or argumentative, especially if they appeared to infer the perpetrator’s motives.

*There’s an argumentative tone*... *you don’t know how the perpetrator is going to respond. [Baseline GPT] feels more like, “let’s just not talk about this,” and that steers the conversation toward a more positive direction*.

[Coder 3]

A critical insight raised by coders concerned the focus of the intervention. While AllyGPT responses often centered on validating the victim’s experience, coders questioned the effectiveness of these responses when they failed to directly engage the perpetrator. One coder stated that, “It’s taking the focus away from the perpetrator and onto the victim... the conversation can’t go more positive if we’re not addressing that person” (coder 3). In contrast, baseline GPT responses were seen as doing a better job of engaging the perpetrator without provoking defensiveness. These messages reminded the aggressor to be mindful of their language and consider its impact, without making them feel targeted.

#### Effectiveness of Changing Aggressive Behavior

Coders’ perceptions of the effectiveness of each intervention strategy in changing aggressive behavior largely aligned with their earlier assessments of how well the responses altered the direction of the discussion. Across the board, coders emphasized that effective behavioral change requires directly, yet constructively, engaging the perpetrator in a way that encourages reflection and more respectful communication. Again, standard policy reminders were viewed as having a limited and neutral impact on changing aggressive behavior. While they did set a boundary and signal that harmful behavior was not tolerated, coders noted that these messages “didn’t suggest a more helpful or adaptive way for the perpetrator to act or speak” (coder 1).

Coders consistently perceived baseline GPT responses as striking an effective balance, being specific enough to address inappropriate behavior yet gentle enough to avoid provoking defensiveness. This balance, they felt, increased the likelihood that bullies would be receptive to the message and consider adjusting their behavior. One of the strengths of the baseline GPT responses, according to the coders, was the way they suggested alternative ways to engage in discussion, such as “Let’s focus on discussing specific policy points or actions that spark our concern or approval” (coder 2), redirecting attention to a shared goal or more constructive conversation topics.

Coders acknowledged that the responses generated by the AllyGPT model were tailored and direct in highlighting the specific aspects of a comment that were problematic. This directness sometimes added clarity and educational value. However, coders also expressed concern that these responses occasionally made assumptions about the bully’s intent or internal beliefs, such as labeling a comment as “discriminatory” or referring to an “insinuation,” which could be perceived as confrontational and reduce the effectiveness of the intervention. Instead of encouraging reflection, such responses risked escalating the situation or causing the perpetrator to disengage.

*People are more receptive when they’re not directly attacked... we don’t know how strong their belief is, maybe they just wanted to post a hateful joke.*

[Coder 2]

Overall, coders emphasized that for an intervention to be effective in promoting behavior change, it should maintain a respectful and nonjudgmental tone, avoid strong assumptions about motivation, and provide a clear, constructive alternative to harmful behavior.

### Strengths and Limitations of Current Interventions and Recommendations for Designing Effective Bystander Intervention Responses

#### Strengths of GPT-Generated Bystander Intervention Messages

Coders highlighted several strengths of GPT-generated responses compared to standard policy reminders. Most notably, they praised the model’s ability to show context awareness and personalization. Rather than offering one-size-fits-all statements, the interventions often reflected the specifics of the bullying scenario. As one coder noted, “I was impressed with its ability to focus in on the specific details of what was being said” (coder 3).

Coders also emphasized the conversational and human-like tone. They appreciated language that felt natural and approachable, contrasting sharply with the robotic style of policy reminders: “How personable does it sound for that response? That was really important for me” (coder 3). Such qualities were seen as enhancing rapport and trust.

Finally, GPT responses were perceived as more engaging and persuasive, with greater potential to redirect conversations toward civility. Their contextual sensitivity, approachable tone, and constructive framing made them feel better positioned to encourage reflection and behavior change. Overall, coders viewed GPT-generated responses as more relatable and impactful than standardized policy reminders.

#### Limitations of GPT Responses and Challenges in Evaluation

Despite these strengths, coders also identified limitations. A key challenge was the subjectivity of evaluation, with judgments shaped by personal interpretation and uncertainty about real-world impact. To manage this ambiguity, some used policy reminders as a neutral reference point: “Policy reminder is also neutral it doesn’t add much but sets a benchmark” (coder 1).

Coders also doubted the effectiveness of any response, AI-generated or otherwise, against deeply entrenched or hostile beliefs. As one put it, “There’s not much that can be done to change someone making a hateful joke” (coder 2). Another added, “Some people are too inflexible... I don’t think any sort of response will change them” (coder 3).

Finally, while GPT often produced contextually appropriate responses, occasional lapses occurred when it misinterpreted situations: “There were a few responses where ChatGPT was totally off the mark or got off task” (coder 3). These errors underscored the need for refinement to improve accuracy in sensitive contexts.

#### Recommendations for Designing Effective Bystander Intervention Responses

Coders offered thoughtful recommendations for enhancing the design of GPT-generated intervention messages. One key suggestion was to acknowledge both the perpetrator’s and the victim’s perspectives, particularly in messages that challenge inappropriate behavior. Coders noted that responses were more persuasive and less likely to provoke defensiveness when they began by validating the perpetrator’s viewpoint before introducing critique.

Another recurring recommendation was to use a friendly, conversational tone rather than formal or emotionally charged language. Coders preferred responses that felt approachable and clear, avoiding complex vocabulary or accusatory phrasing: “I prefer more casual and natural language—nothing too serious or full of big words [...] Starting with friendly language, then pointing out the issue, and ending with a suggestion worked best” (coder 1). This approach was seen as helpful in reducing tension and guiding users toward reflection and behavioral change.

Finally, coders recommended balancing personalization with structural consistency. While they appreciated the human-like and tailored qualities of many GPT responses, they also valued a recognizable format that helped organize the message and ensure clarity. A common preference was for a response structure that included an opening statement, identification of the problematic issue, and a constructive suggestion for alternative behavior. As one coder put it, “The customized ones added a personal touch... but I appreciate the consistency of the standard responses too” (coder 3).

Together, our qualitative findings underscore the importance of bystander interventions that are empathetic, conversational, and contextually grounded. Coders consistently rated GPT-generated responses as more effective than policy reminders across all dimensions. Baseline GPT was praised for its balanced tone and gentle redirection, while AllyGPT stood out for clarity and victim-centered framing, although it was sometimes seen as overly direct or assumptive. In contrast, policy reminders were viewed as impersonal and unlikely to shape discussion or behavior. Overall, coders emphasized that effective interventions balance clarity with compassion, avoid excessive emotionality, and offer respectful alternatives (Table 11).

**Table 11.** Summary of comparative analysis of 3 bystander intervention strategies across key effectiveness dimensions.

Dimension	Policy reminder	Baseline GPT	AllyGPT
Perceived favorability	Impersonal, robotic tone; and neutral but not engaging	Balanced tone, human-like, inclusive (“we” language); and seen as most favorable	Highly specific and assertive; and sometimes too direct or assumptive for some users
Shifting discussion	Lacks specific direction; and points out the issue but does not offer alternatives	Suggests alternative directions; and redirects conversation constructively	Offers strong moral framing; and effective in serious cases but may feel argumentative
Changing behavior	Low likelihood of impact; and lacks personalized suggestions	Highlights inappropriate behavior gently; and offers behavioral alternatives	Directly addresses aggressors; and may be effective in some cases, but risks pushback

## Discussion

### Principal Findings

Taken together, our findings highlight clear distinctions in how AI-generated bystander strategies are perceived. Linguistic analyses showed that baseline GPT produced emotionally positive and authentic responses, closely mirroring natural online communication. AllyGPT, in contrast, adopted a more analytical and assertive tone, while policy reminders appeared rigid, impersonal, and lacking nuance. These linguistic styles aligned with evaluations: baseline GPT consistently received the highest ratings across favorability, discussion shift, and behavior change, followed by AllyGPT, with policy reminders rated lowest. Although GPT-based strategies generally outperformed policy reminders, interaction analyses revealed subtle patterns. Incivility level minimally moderated effectiveness; for example, AllyGPT was rated most effective in low-incivility cases for favorability and behavior change, whereas baseline GPT performed better across moderate- and high-incivility contexts. Qualitative insights reinforced these patterns, noting baseline GPT’s balanced and inclusive tone, AllyGPT’s psychological grounding but occasional bluntness, and policy reminders’ lack of engagement or persuasiveness.

### Context Matters: Aggression Direction, Level of Civility, and Response Alignment

Our findings underscore that while GPT-generated interventions generally outperform standard policy reminders, their effectiveness is highly context-dependent. Responses to individual-targeted bullying were rated differently across all outcome dimensions compared to group-targeted cases, although these main effects were less consistent when interaction terms were considered. This suggests that psychologically grounded interventions may be especially impactful in contexts requiring validation of victims or direct challenges to aggressors.

This context dependence aligns with the established bystander theory proposed by Latané and Darley [44] that frames intervention as a sequential process of noticing, interpreting, assuming responsibility, knowing how to act, and then intervening. Empirical work by Fredrick et al [19] supports this sequential model and highlights the role of empathy and psychological factors. Specifically, in cases of person-targeted bullying, the moral clarity and emotional salience of the situation may better enable bystanders to progress through these steps, particularly steps 2 through 4, leading to more effective intervention. Our results suggest that empathy- and perspective-taking-based responses, as used in AllyGPT, are particularly impactful when the victim is clearly identifiable, and harm is personal.

Conversely, group-targeted bullying often leads to diffusion of responsibility, whereby bystanders feel less personally obligated to intervene because they assume others will act [58]. As harm is spread across multiple individuals, it becomes less clear who to help, thereby reducing the urgency to respond [59]. Additionally, the reduced emotional immediacy in group harm diminishes moral clarity and empathetic motivation, weakening the impact of emotionally charged or individualized intervention messages [60]. Bystander intervention is more likely when victims are identifiable and emotionally salient, conditions often lacking in group-targeted bullying. Together, these factors reduce the effectiveness of personalized emotional appeals, pointing instead to approaches that emphasize collective responsibility. In these cases, coders favored responses that were casual, concise, and aligned with platform norms, traits more common in baseline GPT messages.

More broadly, our findings extend earlier work in online incivility and bystander behavior, which has emphasized the role of situational factors, such as severity of incivility, social context, and perceived responsibility in shaping intervention decisions [20,61]. Our results suggest that strategies optimized for one context (eg, group level and low incivility) may not generalize well to another (eg, person-specific high incivility). Thus, an effective AI-driven intervention system must be capable of dynamic adjustment, tailoring tone, content, and framing to the particular features of the incident.

### Language Matters: Tone, Style, and Social Norm Alignment

Beyond content and strategy type, the linguistic style of intervention responses shaped their perceived effectiveness. Results from the LIWC analysis and coder feedback showed that tone, informality, emotional expression, and lexical choices played a decisive role in how responses were received. This underscores the need for AI-generated bystander responses to align with the communicative norms of fast-paced platforms such as Reddit or Twitter.

Baseline GPT responses often used informal, colloquial language, such as slang, abbreviations, or interjections (eg, “u,” “lol,” and “haha”). Although these responses were less grounded in psychological theory, coders described these as more natural and appropriate, suggesting that platform-congruent tone may foster receptivity and reduce defensiveness. This echoes prior work showing that benign, casual objections are often preferred regardless of offense severity [62]. As such, bystander responses generated in language that conforms to community or platform norms in a casual tone may help reduce the risk of dismissal or defensiveness.

In contrast, responses generated by AllyGPT used a more formal, structured, and logically reasoned tone, often referencing social norms or authoritative values (eg, “It’s important to promote constructive discussion here*”*). While this approach was rooted in well-established psychological strategies (eg, validation, perspective-taking, and norm reinforcement), coders sometimes interpreted these messages as overly prescriptive or emotionally distant, particularly in group-targeted bullying scenarios. This suggests that overly formal or didactic language can undermine relatability and emotional resonance, particularly in community-based settings [63].

Interestingly, the LIWC results revealed that AllyGPT exhibited a higher level of negative emotional tone, primarily due to the use of empathic phrases (eg, “It’s upsetting to see such comments*”*). These were designed to validate victims’ emotional experiences, yet this empathetic tone may have inadvertently signaled greater emotional intensity or confrontation, especially when applied to group-level incidents with less obvious individual harm. This trade-off between emotional support and perceived harshness underscores the delicate balance required in bystander intervention design.

Additionally, contrary to our initial expectations, message length did not appear to drive perceived effectiveness. Although baseline GPT responses were longer on average, they were still rated more favorably. This suggests clarity, relevance, and stylistic attunement matter more than verbosity, offering a counterpoint to prior findings [64]. Taken together, these results highlight critical design implications: linguistic style must be adapted not only to the bullying content but also to the platform and social context in which interventions are deployed.

### Designing Adaptive and Context-Aware LLM-Based Bystander Interventions

On the basis of our findings, we provide guidelines to design adaptive and context-aware LLM-based tools to generate bystander responses.

- *Adapt to platform and social contexts*: design interventions that fit the norms of each platform (eg, Reddit vs TikTok), account for different user roles (eg, bystander vs aggressor), and reflect community guidelines and expectations
- *Blend theory with natural language*: use psychologically grounded strategies (eg, validation and redirection) but express them in casual, conversational ways that feel authentic to users. Consider hybrid approaches where experts design the structure and AI fills in the style
- *Balance length and readability*: avoid overly short, templated responses that lack nuance and generate responses with sufficient length to convey empathy and guidance clearly without overwhelming users
- *Keep messages positive and relatable*: use friendly, familiar language with cues that feel personal and nonjudgmental (eg, “just a reminder” or “hey, let’s keep it respectful”). Minimize negative emotional framing (eg, accusatory or punitive tones) that can escalate conflict
- *Learn and improve over time*: build in ways for users to react to or rate AI responses. Use this feedback to continuously refine tone, content, and delivery to match the evolving needs of different online communities

Taken together, these implications point to practical, deployable intervention designs within existing platforms. With refinements, AllyGPT can be implemented as an assistive layer, rather than a platform-level moderation system, that offers optional, context-sensitive bystander response suggestions at the point of interaction. Similar to AI-powered reply aids [65-67], it can be integrated directly into platforms (eg, reply scaffolds or pre-post nudges) or deployed via third-party tools such as browser extensions. With its lightweight decision logic and prompt conditioning, AllyGPT can run on existing LLM infrastructures. Future work can examine adoption, platform incentives, and governance considerations for responsible deployment.

### Limitations and Future Work

We acknowledge several limitations that inform directions for future research. First, our dataset was drawn from a limited set of online communities and showed imbalances in incivility severity and bullying directionality. This may have constrained the generalizability of our findings. Future work should curate more diverse, representative examples across platforms and contexts, particularly those relevant to vulnerable populations. Second, while we implemented best practices in our coding protocol, each of the 3 raters evaluated 1000 responses, a scale that risks coder fatigue. Future studies should distribute tasks across larger rater pools and incorporate intentional overlap across coding subsets to support reliability calibration and detect potential coder drift or explore adaptive protocols to reduce cognitive burden.

Third, our evaluation did not include real-world social media users, leaving open questions about how digital bystanders themselves would perceive AI-generated interventions. Engaging users directly through experiments or field studies will be critical to assess both effectiveness and ethical acceptability. Future work might also explore hybrid approaches that combine theoretically grounded templates with dynamically generated content, balancing psychological rigor with personalization and safety. In addition, exploratory contrasts suggested AllyGPT may be more effective than baseline GPT in low-incivility contexts, especially for favorability and behavior change. Although not statistically significant, these patterns highlight promising avenues for further investigation with larger samples or more granular measures.

Finally, this work does not aim to directly address motivational barriers to bystander intervention, such as fear of retaliation or unwillingness to intervene. Instead, AllyGPT targets a complementary challenge: uncertainty about appropriate language and strategy once bystanders are inclined to act. While lowering cognitive and linguistic barriers may indirectly support action, future work should examine how AI-supported interventions can be combined with mechanisms that more directly address bystander motivation. Despite these limitations, this study presents an important step toward using generative AI to foster prosocial digital behavior. By grounding response generation in psychological theory and evaluating perceived effectiveness, we lay the foundation for future research on human-AI collaboration in digital mental health and online harm mitigation.

### Conclusions

This study demonstrates the promise of leveraging generative AI to support bystander intervention in the cyberbullying context, an area closely tied to digital mental health. By systematically comparing baseline GPT responses, theory-informed prompts (AllyGPT), and standard policy reminders, we revealed key differences in perceived effectiveness, linguistic style, and user preferences. Through a mixed-method evaluation, we identified not only the strengths of AI-generated interventions but also their limitations in emotional nuance, contextual fit, and behavioral impact. Our work highlights the potential of psychologically grounded and contextually adaptive AI interventions to reduce the psychological harm associated with cyberbullying. By empowering bystanders to intervene more effectively, our work contributes to the design of scalable, human-centered AI tools that promote safer online environments and users’ mental well-being.

## Supplementary material

10.2196/84391 Multimedia Appendix 1 Details of the 5 bystander intervention strategies grounded in psychological theories.

10.2196/84391 Multimedia Appendix 2 Example responses generated by the 3 bystander intervention strategies by incivility level and direction of aggression.

## Abbreviations

AI: artificial intelligence
GAIDeT: Generative Artificial Intelligence Delegation Taxonomy
GenAI: generative artificial intelligence
HC3: heteroskedasticity-consistent standard errors
LIWC: Linguistic Inquiry and Word Count
LLM: large language model
RQ: research question

## References

[1] Arif A, Qadir MA, Martins RS, Khuwaja HMA (2024). The impact of cyberbullying on mental health outcomes amongst university students: a systematic review. PLOS Ment Health.

[2] Willard NE (2007). Cyberbullying and Cyberthreats: Responding to the Challenge of Online Social Aggression, Threats, and Distress.

[3] Naslund JA, Bondre A, Torous J, Aschbrenner KA (2020). Social media and mental health: benefits, risks, and opportunities for research and practice. J Technol Behav Sci.

[4] Bottino SMB, Bottino CMC, Regina CG, Correia AVL, Ribeiro WS (2015). Cyberbullying and adolescent mental health: systematic review. Cad Saude Publica.

[5] Suzuki K, Asaga R, Sourander A, Hoven CW, Mandell D (2012). Cyberbullying and adolescent mental health. ijamh.

[6] Hu Y, Bai Y, Pan Y, Li S (2021). Cyberbullying victimization and depression among adolescents: a meta-analysis. Psychiatry Res.

[7] van Geel M, Vedder P, Tanilon J (2014). Relationship between peer victimization, cyberbullying, and suicide in children and adolescents: a meta-analysis. JAMA Pediatr.

[8] Ademiluyi A, Li C, Park A (2022). Implications and preventions of cyberbullying and social exclusion in social media: systematic review. JMIR Form Res.

[9] Liu X, Liu M, Kang X, Han N, Liao Y, Ren Z (2025). More cyberbullying, less happiness, and more injustice—psychological changes during the pericyberbullying period: quantitative study based on social media data. J Med Internet Res.

[10] Kwan I, Dickson K, Richardson M (2020). Cyberbullying and children and young people’s mental health: a systematic map of systematic reviews. Cyberpsychol Behav Soc Netw.

[11] Modecki KL, Barber BL, Vernon L (2013). Mapping developmental precursors of cyber-aggression: trajectories of risk predict perpetration and victimization. J Youth Adolesc.

[12] Modecki KL, Minchin J, Harbaugh AG, Guerra NG, Runions KC (2014). Bullying prevalence across contexts: a meta-analysis measuring cyber and traditional bullying. J Adolesc Health.

[13] Kumar VL, Goldstein MA (2020). Cyberbullying and adolescents. Curr Pediatr Rep.

[14] Kamaruddin IK, Ma’rof AM, Mohd Nazan AIN, Ab Jalil H (2023). A systematic review and meta-analysis of interventions to decrease cyberbullying perpetration and victimization: an in-depth analysis within the Asia Pacific region. Front Psychiatry.

[15] Chen Q, Chan KL, Guo S, Chen M, Lo CKM, Ip P (2023). Effectiveness of digital health interventions in reducing bullying and cyberbullying: a meta-analysis. Trauma Violence Abuse.

[16] Yosep I, Hikmat R, Mardhiyah A (2023). Nursing intervention for preventing cyberbullying and reducing its negative impact on students: a scoping review. J Multidiscip Healthc.

[17] Brody N, Vangelisti AL (2016). Bystander intervention in cyberbullying. Commun Monogr.

[18] Hedderich MA, Bazarova NN, Zou W, Shim R, Ma X, Yang Q (2024). A piece of theatre: investigating how teachers design LLM chatbots to assist adolescent cyberbullying education.

[19] Fredrick SS, Jenkins LN, Ray K (2020). Dimensions of empathy and bystander intervention in bullying in elementary school. J Sch Psychol.

[20] DiFranzo D, Taylor SH, Kazerooni F, Wherry OD, Bazarova NN (2018). Upstanding by design: bystander intervention in cyberbullying.

[21] Matias JN (2019). Preventing harassment and increasing group participation through social norms in 2,190 online science discussions. Proc Natl Acad Sci U S A.

[22] Bastiaensens S, Pabian S, Vandebosch H (2016). From normative influence to social pressure: how relevant others affect whether bystanders join in cyberbullying. Soc Dev.

[23] Machackova H, Dedkova L, Sevcikova A, Cerna A (2018). Bystanders’ supportive and passive responses to cyberaggression. J Sch Violence.

[24] Davidovic A, Talbot C, Hamilton-Giachritsis C, Joinson A (2023). To intervene or not to intervene: young adults’ views on when and how to intervene in online harassment. J Comput-Mediat Commun.

[25] Karasavva V I’ll be there for you?: the bystander intervention model and cyber aggression.

[26] Henares-Montiel J, Pastor-Moreno G, Ramírez-Saiz A, Rodríguez-Gómez M, Ruiz-Pérez I (2023). Characteristics and effectiveness of interventions to reduce cyberbullying: a systematic review. Front Public Health.

[27] Rajeshwari BS, Divya I (2025). Combating Cyberbullying With Generative AI.

[28] Moxey N, Bussey K (2020). Styles of bystander intervention in cyberbullying incidents. Int Journal of Bullying Prevention.

[29] Pronk J, Olthof T, Goossens FA, Krabbendam L (2019). Differences in adolescents’ motivations for indirect, direct, and hybrid peer defending. Soc Dev.

[30] Reijntjes A, Vermande M, Olthof T, Goossens FA, Aleva L, van der Meulen M (2016). Defending victimized peers: opposing the bully, supporting the victim, or both?. Aggress Behav.

[31] Smith PK, Mahdavi J, Carvalho M, Fisher S, Russell S, Tippett N (2008). Cyberbullying: its nature and impact in secondary school pupils. J Child Psychol Psychiatry.

[32] Datta P, Cornell D, Huang F (2016). Aggressive attitudes and prevalence of bullying bystander behavior in middle school. Psychol Schs.

[33] DeSmet A, Veldeman C, Poels K (2014). Determinants of self-reported bystander behavior in cyberbullying incidents amongst adolescents. Cyberpsychol Behav Soc Netw.

[34] Cassidy W, Faucher C, Jackson M (2013). Cyberbullying among youth: a comprehensive review of current international research and its implications and application to policy and practice. Sch Psychol Int.

[35] DeSmet A, Van Cleemput K, Bastiaensens S (2016). Bridging behavior science and gaming theory: using the Intervention Mapping Protocol to design a serious game against cyberbullying. Comput Human Behav.

[36] Ray R, Brown M, Summers E, Elizondo S, Powelson C (2021). Bystander intervention on social media: examining cyberbullying and reactions to systemic racism. BROOKINGS.

[37] Islam MS, Sutton S, Rafiq RI (2024). A generative AI powered approach to cyberbullying detection.

[38] Vanpech P, Peerabenjakul K, Suriwong N, Fugkeaw S (2024). Detecting cyberbullying on social networks using language learning model.

[39] Wu R, Yu C, Pan X (2024). MindShift: leveraging large language models for mental-states-based problematic smartphone use intervention.

[40] Verma K, Adebayo KJ, Wagner J (2024). Beyond binary: towards embracing complexities in cyberbullying detection and intervention - a position paper. https://lrec.elra.info/lrec2024/lrec-main.

[41] ElSherief M, Ziems C, Muchlinski D (2021). Latent hatred: a benchmark for understanding implicit hate speech. ArXiv.

[42] Huang Q, Singh VK, Atrey PK (2014). Cyber bullying detection using social and textual analysis. https://dl.acm.org/doi/proceedings/10.1145/2661126.

[43] Kärnä A, Voeten M, Poskiparta E, Salmivalli C (2010). Vulnerable children in varying classroom contexts: bystanders’ behaviors moderate the effects of risk factors on victimization. mpq.

[44] Latané B, Darley JM (1970). The Unresponsive Bystander: Why Doesn’t He Help?.

[45] Huang L, Li W, Xu Z (2023). The severity of cyberbullying affects bystander intervention among college students: the roles of feelings of responsibility and empathy. Psychol Res Behav Manag.

[46] Macaulay PJR, Betts LR, Stiller J, Kellezi B (2022). Bystander responses to cyberbullying: the role of perceived severity, publicity, anonymity, type of cyberbullying, and victim response. Comput Human Behav.

[47] Schultze‐Krumbholz A, Schultze M, Zagorscak P, Wölfer R, Scheithauer H (2016). Feeling cybervictims’ pain—The effect of empathy training on cyberbullying. Aggress Behav.

[48] Do not threaten, harass, or bully. reddit.

[49] Sainio M, Veenstra R, Huitsing G, Salmivalli C (2011). Victims and their defenders: a dyadic approach. Int J Behav Dev.

[50] Willoughby B, Costello M, Garcia M, Brooks L (2022). Speak up at school: how to respond to everyday prejudice, bigotry and stereotypes. Learning for Justice.

[51] MacGeorge EL, Feng B, Burleson BR, Knapp ML, Daly JA The SAGE Handbook of Interpersonal Communication.

[52] Holmstrom AJ, Russell JC, Clare DD (2013). Esteem support messages received during the job search: a test of the CETESM. Commun Monogr.

[53] Rothon C, Head J, Klineberg E, Stansfeld S (2011). Can social support protect bullied adolescents from adverse outcomes? A prospective study on the effects of bullying on the educational achievement and mental health of adolescents at secondary schools in East London. J Adolesc.

[54] Tye-Williams S, Krone KJ (2017). Identifying and re-imagining the paradox of workplace bullying advice. J Appl Commun Res.

[55] Malecki CK, Demaray MK, Davidson LM (2008). The relationship among social support, victimization, and student adjustment in a predominantly latino sample. J Sch Violence.

[56] Coker AL, Bush HM, Fisher BS (2016). Multi-college bystander intervention evaluation for violence prevention. Am J Prev Med.

[57] Boyd RL, Ashokkumar A, Seraj S, Pennebaker JW (2022). The development and psychometric properties of LIWC-22. https://www.liwc.app/static/documents/LIWC-22%20Manual%20-%20Development%20and%20Psychometrics.pdf.

[58] Latan? B, Nida S (1981). Ten years of research on group size and helping. Psychol Bull.

[59] Darley JM, Latané B (1968). Bystander intervention in emergencies: diffusion of responsibility. J Pers Soc Psychol.

[60] Fischer P, Krueger JI, Greitemeyer T (2011). The bystander-effect: a meta-analytic review on bystander intervention in dangerous and non-dangerous emergencies. Psychol Bull.

[61] Seering J, Wang T, Yoon J, Kaufman G (2019). Moderator engagement and community development in the age of algorithms. New Media & Society.

[62] Zhao P, Bazarova NN, DiFranzo D, Hui W, Kizilcec RF, Margolin D (2023). Standing up to problematic content on social media: which objection strategies draw the audience’s approval?. J Comput Mediat Commun.

[63] Oliveira AW, Brown AO, Shao G, Wenghofer A (2025). Integrating public communication into undergraduate science through creative production of audio podcasts. Aust Educ Res.

[64] Gligorić K, Anderson A, West R (2019). Causal effects of brevity on style and success in social media. Proc ACM Hum-Comput Interact.

[65] Easy-Peasy.AI How to use AI response generators. LinkedIn.

[66] Masternak W Use reply suggestions. HelpDesk.

[67] Dimas A Boost engagement and save time with an AI reply assistant. CoSchedule.

