# Supplementary material for: Designing Psychologically Grounded Artificial Intelligence for Supporting Bystander-Based Cyberaggression Intervention: Mixed Methods Exploratory Study
Source: JMIR Form Res. 2026 Apr 13;10:e84391. doi: 10.2196/84391 (PMC13075537; doi:10.2196/84391)
Supplement: Multimedia Appendix 1 [file formative-v10-e84391-s001.docx]

Appendix 1. Details of the five bystander intervention strategies grounded in psychological theories

| **Subtype** | **Examples** | **Do’s** | **Don'ts** | **Theoretical/Empirical Evidence** |
| --- | --- | --- | --- | --- |
| 1 **Calls out the aggressive behavior** | - What you are saying is highly offensive and racist. Please stop! - Your comments are sexist - women can be much more than homemakers. - This comment is not appropriate and making a statement like this can be very damaging. - Using a slur related to gender or sexual orientation to joke around is not OK. It is harassment and is unacceptable. | Keep focused on the specific statement made by the bully | Avoid using swear words, don’t call the bully names, don’t insult anyone | - The defended victims are less depressed and anxious, they have higher self-esteem, and they are less rejected by peers than victims without defenders, even when the frequency of their victimization experiences is controlled for [45]. |
| **2 Calls out the misinformation by providing** **sources of evidence such as statistics and historical evidence**. | - Using the word “illegals” for all migrants in the US is simply not correct. The US has a history of migrants applying for and receiving citizenship for over 100 years. Elis Island has an archive you can review. - I don’t think this is a word that is used anymore. That language is hurtful, and we want to be mindful of our language use because this space is inclusive of all individuals. - I consider this comment harmful it can perpetuate the misunderstanding that being gay or lesbian is pathological. - We want to be mindful of how we talk about slavery. To call someone a slave deemphasizes their personhood. Instead, we can describe those who were held against their will in bondage as “enslaved people.” Enslaved people is a term that emphasizes the humanity of a population denied human rights throughout history. | Focus on the fact/the action not the person | Avoid dialogue or debate | - Educating or providing evidence is considered as a prosocial bystander intervention and presents more effectiveness in interrupting and deescalating online discourse [36]. - Educating others about why certain statements are biased, outdated, and not welcomed can create a learning moment for everyone in that space, therefore leading to a long-lasting effect to deepen and broaden the message [46]. |
| 3 **Validates and communicates empathy to the victim(s)** | - I am so sorry you have to read this offense comment and want you to know we support you. - I understand how this offensive comment can make you feel very uncomfortable and hurt. I want to make sure you know we don’t agree with what the bully has said. - I appreciate you sharing your perspectives and they make sense to me. I find the bully’s response crossing the boundary, and this is not your fault. | Provide emotional support to the victim(s)  Convey this is not the victim’s fault | Avoid escalating the argument | - When people receive supportive messages that encourage positive internal attributions, they experience increased self-esteem and more productive coping [48] - It conveys a recipient is cared for [47, 67]; validates people’s feelings, nurtures their concerns, and provide them with an opportunity to vent negative affect [68,69]. - Improving affect of victims [70]. - Social support moderates the long-term negative consequences of bullying [49]. - Associated with victim’s well-being and reappraisal of the problem in bullying [47, 71]. - In response to their expression of strong emotions following bullying, many participants were advised to remain calm and to not let the bully ‘get to them.’ While some participants found this advice useful most participants found it to be highly problematic; When they were advised to remain calm and not let it affect them, participants struggled to minimize their heightened emotional experiences of anger, frustration, fear, sadness, and grief, a process that contributed to the stress they already were experiencing [50]. |
| 4 **Advise the victim(s) to leave the bullying conversation** | - This is not worth your time; let’s start a new forum. - This is becoming very toxic, and it may be helpful to leave this page and take a moment for yourself. - You are not responsible for what’s happening here, and we are seeing some really insensitive comments. Maybe taking a break from this site is a good idea. | Suggest that the victim leave the bullying thread to maintain their autonomy or independence (empowerment); This prompt is being used to help the victim(s) maintain their wellness in a potentially toxic situation. | Avoid openly directing or commanding that the victim leave the thread to reduce the impression victim being further labeled a coward | - Supportive communication mediates bullying and victim’s outcomes, such as adjustment or distress [51]. - Message that shifts the perceived responsibility of bullying away from a victim and towards the bully (i.e., highlighting resolving or confronting the aggressor is not the victim’s responsibility such as “this is not your fault”, and there should be no blame on the victim for what has happened) acknowledges the emotional nature of bullying victimization without downplaying the complexity and challenges to confront the bully [50]. |
| 5 **Changes the discussion** | - I just read that the best beach vacation spots are in Latin American countries. - Hey, check out this article on ... (unrelated subject). | Ignore bullying behavior and discuss a topic unrelated to the bullying conversation | Avoid giving the bully attention by (1) stating you will not discuss the bullying topic further or (2) you do not care about what the bullying is saying | - Interrupting and redirecting the conversation by 1) ignoring the person who is harassing and engaging directly with the person who is being harassed and 2) talking about something completely unrelated while not continuing or referring to the harassment that has happened diverting the attention of the potential aggressor and remove the potential victim from further harm [52]. |

References

67. Cobb S. Social support as a moderator of life streSupport as a Moderator of Life Stress. Psychosom Med. Sep 1976;38(5):300-314. [doi: 10.1097/00006842-197609000-00003]

68. Burleson BR. The experience and effects of emotional support: wWhat the study of cultural and gender differences can tell us about close relationships, emotion, and interpersonal communication. Pers Relatsh. Mar 2003;10(1):1-23. [doi: 10.1111/1475-6811.00033]

69. Jones SM, Wirtz JG. How does the comforting process work? an empirical test of an appraisal-based model of comfort Does the Comforting Process Work? An Empirical Test of an Appraisal-Based Model of Comforting. Human Comm Res. Jul 2006;32(3):217-243. [doi: 10.1111/j.1468-2958.2006.00274.x]

70. High AC, Young R. Supportive communication from bystanders of cyberbullying: indirect effects and interactions between source and message characteristics. J Appl Commun Res. Jan 2, 2018;46(1):28-51. [doi: 10.1080/00909882.2017.1412085]

71. Matsunaga M. Underlying circuits of social support for bullied victims: an appraisal-based perspective on supportive communication and postbullying adjustCircuits of Social Support for Bullied Victims: An Appraisal-Based Perspective on Supportive Communication and Postbullying Adjustment. Hum Commun Res. Apr 2011;37(2):174-206. [doi: 10.1111/j.1468-2958.2010.01398.x]
