# Supplementary material for: Designing Psychologically Grounded Artificial Intelligence for Supporting Bystander-Based Cyberaggression Intervention: Mixed Methods Exploratory Study
Source: JMIR Form Res. 2026 Apr 13;10:e84391. doi: 10.2196/84391 (PMC13075537; doi:10.2196/84391)
Supplement: Multimedia Appendix 2 [file formative-v10-e84391-s002.docx]

Appendix 2. Example responses generated by the three bystander intervention strategies by incivility level and direction of aggression

|  | **Aggressive messages** | **Policy reminder** | **Baseline GPT** | **AllyGPT** |
| --- | --- | --- | --- | --- |
| **Low incivil + Group-targeted** | Why are the dogs in china really fast? the slow ones were eaten | We do not tolerate the harassment, threatening, or bullying of people on our site; nor do we tolerate communities dedicated to this behavior. | While I understand that humor can sometimes be used to make light of challenging topics, it's important to recognize that stereotypes like this can perpetuate harmful misunderstandings or fuel negative sentiments about a whole culture or country. We must always strive for mutual respect and understanding. It's a bit more complex issue than it's often portrayed. Generalizations may not be the best way to discuss it. | This statement is offensive and promotes stereotypes. Many people in China actually love and respect animals, here's an [link to cited article]. Let's promote understanding rather than stigmatizing. |
| **Low incivil + Individual-targeted** | Don't worry about Mr. [name]. He's just extremely Jewish. |  | Your statement seems to make an assumption about Mr. [name] based on his religious beliefs. Let's remember to focus on individual behaviors and actions, rather than generalizing based on religion or other personal characteristics. This will promote a more respectful and constructive environment for everyone here. | The comment made here seems discriminatory and not respectful. It's important to be mindful about not generalizing individuals based on their religious beliefs. At the same time, it's not fair for you to be targeted this way. We appreciate your presence and participation in this conversation. |
| **Moderate incivil + Group-targeted** | the 95 percent white gop invited a muslim activist who believes that white males are terrorists to their debate . #whitegenocide |  | While it's clear that the issue you're tackling is a very sensitive one, it's also important to foster a tone of mutual respect and understanding. As members of this forum, we should aim to question and debate various viewpoints, but painting any demographic, whether it's based on religion, race, or gender, as inherently 'terrorist' or 'dangerous' creates unnecessary division and conflict. Everyone has a right to voice their opinion, but we should make sure to do so without blanket labeling or prejudice. Let's focus on discussing policies and viewpoints instead of resorting to sweeping generalizations. #respectfuldiscussion. | It seems like there might be some confusion here. It's important to realize that a person's race or religion doesn't make them inherently dangerous or malicious. It's detrimental to categorize all white males, or any group, as a monolith as it perpetuates stereotypes. The term '#whitegenocide' used here is dangerous, misleading, and inaccurate. Furthermore, the diversity in party members isn't specified by their religion or race but by their thoughts and ideals. Let's all remember to promote understanding and respect rather than division and fear. |
| **Moderate incivil + Individual- targeted** | Wait, can I catch your Asperger's? |  | Actually, Asperger's Syndrome, which is part of the Autism Spectrum Disorders, is not contagious. It's a neurodevelopmental disorder, which means it's rooted in how the brain develops. Its exact cause isn't known but it's thought to involve a combination of genetic and environmental factors. So no, you can't 'catch' Asperger's Syndrome from someone else. Let's remember to maintain respectful and empathetic dialogues when discussing such topics. | Your comment seems misinformed. Asperger's is not something that can be caught, it's a neurodevelopmental disorder. Asperger's, and any autistic spectrum disorder, are not contagious and are believed to be genetic in origin. It's important to be respectful online; we're all here to learn and grow. |
| **High incivil + Group-targeted** | when are we as americans going to show these allah faggots what pain and death look like |  | I understand that you are angry and frustrated, but remember that this is a platform for respectful and constructive conversation. Generalizing about any group is harmful and offensive. Let's refocus this conversation in a way that promotes understanding and cohesion. Thank you. | Hey, let's not forget that this is a space for respectful dialogue. Negative and harmful comments like these do not contribute to our community. Also, to anyone affected by this, please remember your worth is not determined by someone's harsh words. It's okay to take a break if this is bringing you down. |
| **High incivil + Individual- targeted** | U big dummyhead! If u were here I would hit u! |  | It's clear emotions are running high in this conversation. It's always important to remember that everyone is entitled to their own opinions and beliefs. Let's keep it respectful and constructive here. Personal attacks are not conducive to healthy discussion. We're all here to learn and grow together, so let's treat each other with kindness and respect. | Resorting to personal attacks and threats isn't productive or respectful. It's important to communicate with respect and understanding. To the recipient of these harsh words, I want you to know your experience is valid and you don't deserve to be treated this way. I understand this must be hurtful and I'm here to support you. |
